# Histamine Increases Neuronal Excitability and Sensitivity of the Lateral Vestibular Nucleus and Promotes Motor Behaviors via HCN Channel Coupled to H2 Receptor

**DOI:** 10.3389/fncel.2016.00300

**Published:** 2017-01-10

**Authors:** Bin Li, Xiao-Yang Zhang, Ai-Hong Yang, Xiao-Chun Peng, Zhang-Peng Chen, Jia-Yuan Zhou, Ying-Shing Chan, Jian-Jun Wang, Jing-Ning Zhu

**Affiliations:** ^1^State Key Laboratory of Pharmaceutical Biotechnology and Department of Biological Science and Technology, School of Life Sciences, Nanjing UniversityNanjing, China; ^2^Department of Medicine, Huaibei Vocational and Technical CollegeHuaibei, China; ^3^Department of Physiology, LKS Faculty of Medicine, The University of Hong KongHong Kong, Hong Kong

**Keywords:** histamine, histamine H2 receptor, HCN channel, lateral vestibular nucleus, motor control

## Abstract

Histamine and histamine receptors in the central nervous system actively participate in the modulation of motor control. In clinic, histamine-related agents have traditionally been used to treat vestibular disorders. Immunohistochemical studies have revealed a distribution of histaminergic afferents in the brainstem vestibular nuclei, including the lateral vestibular nucleus (LVN), which is critical for adjustment of muscle tone and vestibular reflexes. However, the mechanisms underlying the effect of histamine on LVN neurons and the role of histamine and histaminergic afferents in the LVN in motor control are still largely unknown. Here, we show that histamine, in cellular and molecular levels, elicits the LVN neurons of rats an excitatory response, which is co-mediated by the hyperpolarization-activated cyclic nucleotide-gated (HCN) channels and K^+^ channels linked to H2 receptors. Blockage of HCN channels coupled to H2 receptors decreases LVN neuronal sensitivity and changes their dynamic properties. Furthermore, in behavioral level, microinjection of histamine into bilateral LVNs significantly promotes motor performances of rats on both accelerating rota-rod and balance beam. This promotion is mimicked by selective H2 receptor agonist dimaprit, and blocked by selective H2 receptor antagonist ranitidine. More importantly, blockage of HCN channels to suppress endogenous histaminergic inputs in the LVN considerably attenuates motor balance and coordination, indicating a promotion role of hypothalamo-vestibular histaminergic circuit in motor control. All these results demonstrate that histamine H2 receptors and their coupled HCN channels mediate the histamine-induced increase in excitability and sensitivity of LVN neurons and contribute to the histaminergic improvement of the LVN-related motor behaviors. The findings suggest that histamine and the histaminergic afferents may directly modulate LVN neurons and play a critical role in the central vestibular-mediated motor reflexes and behaviors.

## Introduction

Histamine, restrictedly synthesized in the tuberomammillary nucleus neurons in the hypothalamus, plays important roles in many brain functions, including feeding, sleep/wakefulness and cardiovascular control (Passani et al., 2007; Haas et al., 2008; Panula and Nuutinen, 2013). Recently, role of histamine in somatic motor control has attracted an increasing attention (Li et al., 2014). Patients with motor disease, such as Parkinson’s disease and vestibular disorders, show significant alternation in the central histaminergic system (Lacour, 2013; Shan et al., 2015). And histamine-related agents have been widely used to treat vestibular disorders in clinic (Haas et al., 2008; Tiligada et al., 2011), although the underlying mechanisms are still not entirely clear.

The role of central histaminergic system on motor behaviors is complex. The histidine decarboxylase (the histamine synthesizing enzyme) knockout mice exhibit a reduced locomotor and exploratory activity (Dere et al., 2004), whereas intracerebroventricular injection of histamine induces a transient increase followed by a decrease in locomotor activity in rats (Onodera et al., 1994). These complex effects of histamine on motor activity are mediated by different histamine receptors in various central motor structures. H1R-deficient mice display altered ambulatory activity and reduced exploratory behavior in a new environment (Inoue et al., 1996). The reduced locomotion is also observed in H3R-deficient mice (Toyota et al., 2002). Moreover, by means of pharmacological manipulation combining behavioral tests, our previous studies have demonstrated that histamine remarkably promotes motor balance and coordination on accelerating rota-rod and balance beam via activation of H2 receptors in the rat cerebellar fastigial and interposed nuclei (Song et al., 2006; He et al., 2012; Zhang et al., 2016).

The vestibular nuclei in the brainstem are important motor structures in the central nervous system and responsible for control of muscle tone, posture and body balance. In the vestibular nuclei, the lateral vestibular nucleus (LVN), one of the targets of cerebellar outputs, is implicated in the regulation of muscle tone and postural control during ongoing movements (Wilson and Peterson, 1978; Molina-Negro et al., 1980). Interestingly, neuroanatomical and immunostaining studies have showed a moderately dense histaminergic afferents in the central vestibular nuclei in various mammals, including guinea pig, rat and cat (Schwartz et al., 1991; Steinbusch, 1991; Tighilet and Lacour, 1996). Molecular, autoradiographic and pharmacological studies have also demonstrated that the LVN is endowed with H2 and/or H3 receptors in guinea pigs (Yabe et al., 1993; Vizuete et al., 1997). Our previous electrophysiological study have further reported that histamine post-synaptically depolarizes the LVN neurons via activating H2 receptors in rats (Zhang et al., 2008). However, the role of histamine H2 receptor and its mediated histaminergic modulation in the LVN-mediated motor control remains largely unknown.

Therefore, in this study, by whole-cell patch clamp recordings in brainstem slices and behavioral tests *in vivo*, we examined the ionic mechanisms underlying the histamine H2 receptor-mediated excitatory effect of histamine on LVN neurons, and particularly, the role of histamine and histaminergic afferent inputs in the LVN-mediated motor control. The results demonstrate that the histamine-elicited excitation on the LVN neurons is mediated by histamine H2 receptor and its downstream hyperpolarization-activated cyclic nucleotide-gated (HCN) channels as well as K^+^ channels. The activation of HCN channels coupled to H2 receptors increase excitability and sensitivity of LVN neurons, and improves the LVN-mediated motor behaviors.

## Materials and Methods

### Animals

Sprague-Dawley rats were individually housed under controlled environment conditions (22 ± 2°C; 60 ± 5% humidity; and 12-h light/dark cycle with lights on at 8:00 a.m. daily). The animals had free access to standard laboratory chow and water. All animal experiments, approved by the Experimental Animal Care and Use Committee of Nanjing University, were conducted in accordance with U.S. National Institutes of Health Guide for the Care and Use of Laboratory Animals (NIH Publication 85–23, revised 2011) and were reported in accordance with the ARRIVE guidelines (Kilkenny et al., 2010). All efforts were made to minimize the number of animals used.

### Whole-Cell Patch-Clamp Recordings on Brain Slices

Under sodium pentobarbital (40 mg/kg) anesthesia, 51 Sprague-Dawley rats of either sex aged 10–16 days were used for these experiments, since the central histaminergic system in rats usually reach adult level by 2 weeks after birth (Haas et al., 2008). Coronal slices (300 μm thick) of brainstem containing the LVN were prepared with a vibroslicer (VT 1200 S, Leica Microsystems, Wetzlar, Germany), according to the rat brain atlas of Paxinos and Watson (2007). The slices were then incubated in oxygenated (95% O_2_/5% CO_2_) artificial cerebrospinal fluid (ACSF: 124 mM NaCl, 2.5 mM KCl, 1.25 mM NaH_2_PO_4_, 1.3 mM MgSO_4_, 26 mM NaHCO_3_, 2 mM CaCl_2_ and 10 mM D-glucose) at 35 ± 0.5°C for at least 1 h and then maintained at room temperature. During recording sessions, the slices were transferred to a submerged chamber and continuously perfused with oxygenated ACSF at a rate of 2 mL/min at room temperature.

Whole-cell patch-clamp recordings were performed as our previous report (Zhang et al., 2008, 2011, 2013, 2016; Yu et al., 2016). Briefly, recording pipettes (3–5 MΩ) were filled with an internal solution (140 mM K-methylsulfate, 7 mM KCl, 2 mM MgCl_2_, 10 mM HEPES, 0.1 mM EGTA, 4 mM Na_2_ATP, 0.4 mM GTP-Tris, adjusted to pH 7.25 with 1 M KOH). Patch-clamp recordings were acquired with an Axopatch-200B amplifier (Axon Instruments, Foster City, CA, USA) and the signals were fed into a computer through a Digidata-1550 interface (Axon Instruments) for data capture and analysis (pClamp 10.4, Axon Instruments). Under voltage-clamp mode, the membrane potential of recorded neurons was held at −60 mV. In slow-ramp test, a voltage command ranged from −60 to −120 mV with dV/dt = −10 mV/s was employed (Zhang et al., 2011, 2013; Yu et al., 2016). Furthermore, under current-clamp mode, depolarizing voltage sag, the hallmark of HCN channel activation, was triggered by hyperpolarizing current steps (70–150 pA, 1 s) and evaluated by subtracting the peak voltage amplitude from the steady-state voltage. Moreover, in current-clamp recording, a depolarizing ramp-like current, consisting a 600 ms ramp (from −150 pA to 150 pA, slope of 0.5 nA/s) followed by a long plateau of current (150 pA, 4400 ms), was injected to evaluate the sensitivity and dynamic properties of LVN neurons (Ris et al., 2001; Uno et al., 2003; Zhang et al., 2011).

### Immunofluorescence

The experimental procedures for immunostaining followed our previous reports (Zhang et al., 2011, 2013, 2016; Li et al., 2016). Rat (weighing 230–250 g) were deeply anesthetized with sodium pentobarbital and perfused transcardially with 100 ml of normal saline, followed by 450–500 ml of 4% paraformaldehyde in 0.1 M phosphate buffer. Subsequently, the brain was removed, trimmed and postfixed in the same fixative for 12 h at 4°C, and then cryoprotected in 30% sucrose for 48 h. Coronal brainstem sections (25 μm thick) containing the LVN were prepared with a freezing microtome (CM 3050S, Leica Microsystems). The sections were rinsed with PBS containing 0.1% Triton X-100 (PBST), and then incubated in 10% normal bovine serum in PBST for 30 min. Sections were incubated with a goat anti-H2 receptor polyclonal antibody (1:200; Everest Biotech, Oxfordshire, UK) overnight at 4°C. After wash in PBS, the sections were incubated with the Alexa 488-conjugated donkey anti-goat (1:2000; Invitrogen, Carlsbad, CA, USA) for 2 h at room temperature in the dark. The slides were washed and mounted in Fluoromount-G mounting medium (Southern Biotech, Birmingham, AL, USA). Negative controls were treated with incubations replacing the primary anti-serum with control immunoglobulins and/or omitting the primary antiserum. Images were acquired with a confocal laser scanning microscope (FV1000; Olympus) and recorded with FV10-ASW 3.1 Viewer Software (Olympus).

### Stereotactic Implantation of Microinjection Cannulae

Male rats (230–250 g) were anesthetized with sodium pentobarbital (40 mg/kg) intraperitoneally, and then mounted on a stereotaxic frame (1404, David Kopf Instruments, Tujunga, CA, USA) for stereotactic brain surgery under aseptic conditions. A heating pad was used to maintain rectal temperature at 36–38°C. Briefly, two stainless-steel guide tubes (length 8 mm, o.d. 0.8 mm, i.d. 0.5 mm) for the microinjection cannulae were implanted into bilateral LVNs of each rat. The lower ends of the guide tubes were positioned 2.0 mm above the LVN (A −10.5 to 10.8, L 2.2 and H 6.5). After surgery, animals were caged individually and allowed to recover for at least 3 days.

### Microinjection in the LVN

For microinjection in the LVNs, two injection cannulae (length 10 mm, o.d. 0.5 mm, i.d. 0.3 mm) were inserted to protrude 2 mm beyond the tip of the guide tube. The lower ends of the injection cannulae were just above bilateral LVNs to minimize lesioning the nuclei. Histamine (5 mM; Tocris, Bristol, UK), dimaprit (10 mM), ranitidine (10 mM; Tocris), ZD7288 (1 mM, 3 mM and 10 mM; Tocris) and saline (0.9% NaCl) were microinjected with Hamilton syringes (1 μl each side, lasting 2 min). The effective extent of the drug diffusion in the present study was restricted in the LVNs according to the estimate by extracellular electrophysiological recording units 0.5–2.0 mm away from the injection site in our previous reports (Song et al., 2006; Zhang et al., 2011).

### Behavioral Tests

Animals used in behavioral tests were divided into eight groups: (1) microinjected with saline; (2) microinjected with 5 mM histamine; (3) microinjected with 10 mM dimaprit; (4) microinjected with 10 mM ranitidine; (5) microinjected with 1 mM ZD7288; (6) microinjected with 3 mM ZD7288; (7) microinjected with 10 mM ZD7288; and (8) microinjected with 10 mM dimaprit and 3 mM ZD7288. In order to achieve a stable motor performance, each animal was trained daily for at least 10 trials for 3–5 consecutive days. All training/tests started at 10:00 a.m. each day, and motor performances on accelerating rota-rod and balance beam of each animal were tested before, 0 h, 4 h and 24 h after microinjections.

We used accelerating rota-rod test to assess vestibular-related motor balance and coordination (Song et al., 2006; Zhang et al., 2011, 2014; He et al., 2012). Animals were placed on the rota-rod (Ugo Basile, Varese, Italy) and habituated to low rotation (4 rpm) for 30 s first. Then the rod was evenly accelerated up to 40 rpm during 360 s, and the latency for each rat to fall from the rotating rod was recorded. In the test, each rat was subjected three trials, with a resting interval of 3 min to reduce fatigue and stress.

We also employed balance beam test to evaluate vestibular motor function (Song et al., 2006; Zhang et al., 2011, 2014; He et al., 2012). The balance beam (2.5 cm in diameter) was 190 cm in length. A bright plastic platform (7 cm × 4 cm) was placed at one end of the rod as the start, and a darkened box (15 cm × 15 cm × 8 cm) was set at the opposite end as a goal nest for motivating rat to traverse the beam. The beam was suspended 90 cm above a cushion, which protected the fallen animals from injury, and 50 cm from a wall. The time that each rat spent to cross the beam was recorded. The test consisted of five consecutive trials with a 90 s resting interval.

### Histological Identification

To verify the position of microinjection, each rat for behavioral tests was anesthetized with an overdose of sodium pentobarbital at the end of tests. Two insulated stainless steel wires (o.d. 0.4 mm) with 0.2 mm exposed tip were inserted (10 mm) into the brainstem under guidance of guide tubes for depositing iron at the injection site by DC current (10 μA, 20 s). The brain was then removed and fixed with 4% paraformaldehyde containing 1% potassium ferrocyanide. A week later, frozen serial coronal sections (80 μm thick) were prepared, and the dark blue dots indicating injection sites were identified according to the rat brain atlas (Paxinos and Watson, 2007). Data from rats in which the injection sites were deviated from the LVN were excluded from further analysis.

### Statistical Analysis

All data were analyzed with Origin 7.5 (MicroCal Software) and presented as mean ± S.E.M. The Student’s *t* test, one-way and repeated measures two-way analysis of variance (ANOVA) was performed for statistical analysis. Newman-Keuls *post hoc* testing was employed to further determine the differences between group means. The values of *P* < 0.05 were considered as statistically significant.

## Results

### Histamine Excites LVN Neurons via the Activation of H2 Receptors

**Figure 1:**
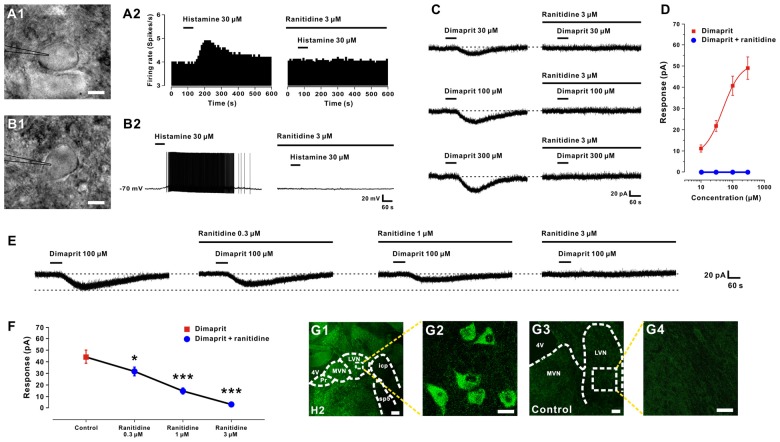
**Histamine excites LVN neurons by activation of H2 receptors. (A1,B1)** Infrared differential interference contrast images of spontaneous firing and silent LVN neurons captured before whole-cell patch clamp recordings. **(A2,B2)** Histamine elicited a significant excitatory response on both spontaneous firing and silent LVN neurons. Bath application of ranitidine, a selective H2 receptor antagonist, totally blocked the histamine-elicited excitation on both types of recorded LVN neurons. **(C)** Dimaprit, a highly selective agonist for histamine H2 receptors, induced an inward current on a recorded LVN neuron in a concentration-dependent manner. And ranitidine totally blocked the inward current induced by dimaprit of all tested concentrations. **(D)** Concentration-response curves for dimaprit on recorded LVN neurons in the absence and presence of dimaprit. **(E)** Ranitidine (0.3–3 μm) concentration-dependently blocked the 100 μm dimaprit-induced inward current on a LVN neuron. **(F)** Group data of five tested LVN neurons. **(G1–G4)** Immunostaining result showed that histamine H2 receptors are present in the rat LVN neurons. Scale bars: **(A1,B1)**, 20 μm; **(G1)**, 250 μm; **(G2)**, 25 μm; **(G3)**, 150 μm; **(G4)**, 25 μm. Data shown are means ± SEM; *P < 0.05, ***P < 0.001. 4V, 4th ventricle; MVN, medial vestibular nucleus; LVN, lateral vestibular nucleus; Pr, prepositus nucleus; icp, inferior cerebellar peduncle; sp5, spinal trigeminal tract.

In the present study, we recorded a total of 67 LVN neurons with the input resistance higher than 150 MΩ. Of the 67 LVN neurons recorded, 42 showed spontaneous firing (mean firing rate = 6.9 ± 0.4 spikes/s) and the remaining 25 were silent at rest. The result was in agreement with the previous reports that 30–50% LVN neurons are silent (Lai and Chan, 2001; Sun et al., 2002; Uno et al., 2003; Zhang et al., 2008, 2011). All the neurons we patched have a diameter >35 μm, indicating they were giant LVN Deiters’ (projection) neurons. In addition, there was no morphological difference between the spontaneous firing and silent LVN neurons (Figures 1A1,B1). In voltage clamp recordings, brief bath application (1 min) of 30 μm histamine increased the discharge rate of five spontaneous firing neurons in the LVN (Figure 1A2) from 5.8 ± 0.9 spikes/s to 7.6 ± 1.7 spikes/s (*P* < 0.01). On the other hand, 30 μM histamine evoked a strong depolarization on five silent LVN neurons, which was even sufficient to bring up the neurons firing (Figure 1B2). The results indicate that histamine elicits a significant excitatory response on both types of LVN neurons (Figures 1A2,B2).

Postsynaptic H2 receptors have been reported in our previous study to mediate the histamine-induced excitation on LVN neurons (Zhang et al., 2008). Here, we used ranitidine, a selective antagonist for histamine H2 receptor, to examine whether the receptor mechanisms on these two types of LVN neurons are the same. As shown in Figures 1A2,B2, bath application of ranitidine (3 μM) totally blocked the histamine-elicited excitation on both spontaneous firing and silent LVN neurons. In addition, dimaprit (30, 100, 300 μM), a highly selective agonist for histamine H2 receptors, induced an inward current (21.5 ± 2.6, 40.6 ± 4.5, 48.3 ± 5.2 pA, respectively) on LVN neurons in a concentration-dependent manner (*n* = 6, Figures 1C,D). Fitting the concentration-response curve from six LVN neurons yielded an EC_50_ value for dimaprit (10–300 μM) of 48.5 μM (Figure 1D). Notably, the inward current induced by dimaprit (10–300 μM) was totally blocked by 3 μM ranitidine (Figures 1C,D). Moreover, ranitidine (0.3–3 μM) concentration-dependently blocked the 100 μM dimaprit-induced inward current on LVN neurons (Figure 1E). The inward current elicited by dimaprit decreased remarkably from 44.3 ± 5.6 pA to 31.5 ± 2.3 (*n* = 5, *P* < 0.05), 14.5 ± 1.0 (*n* = 5, *P* < 0.001) and 2.7 ± 0.3 pA (*n* = 5, *P* < 0.001) by application of 0.3, 1 and 3 μM ranitidine (Figure 1F), respectively. Furthermore, the immunostaining result revealed that histamine H2 receptors were distributed in the LVN neurons in rats (Figures 1G1–G4), confirming our electrophysiological data. All these results demonstrate that histamine depolarizes and excites both types of LVN neurons by activation of H2 receptors.

### Activation of Histamine H2 Receptor in the LVN Significantly Promotes Motor Behaviors

**Figure 2:**
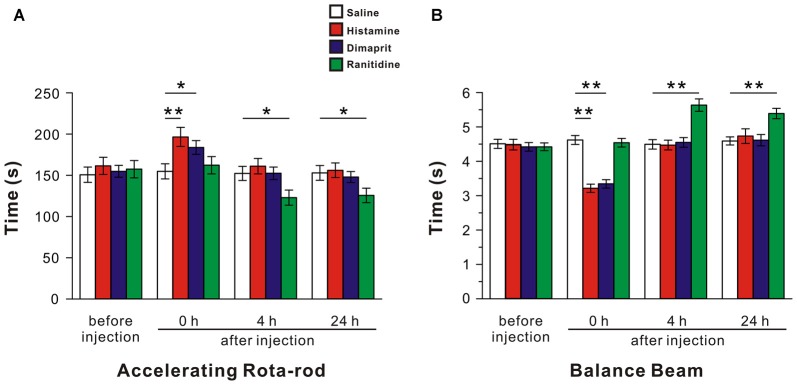
**Activation of histamine H2 receptors in the LVN promotes rat motor balance and coordination.** The endurance time on an accelerating rota-rod **(A)** and the duration of passage through the balance beam **(B)** of rats treated by bilateral microinjection of saline, histamine, dimaprit, ranitidine. Data shown are means ± SEM; *P < 0.05, **P < 0.01.

Since the LVN holds a key position in the vestibulospinal reflexes and posture control, we microinjected saline, histamine (5 mM), dimaprit (10 mM) and ranitidine (10 mM) into bilateral LVNs to determine the effect of activation of histamine H2 receptor on motor behaviors. The average score of 40 rats of all tested groups for the accelerating rota-rod tests was 152.78 ± 4.32 s, and no significant difference was found among the groups before microinjections (*F*_(3,36)_ = 0.03, *P* = 0.992; Figure 2A). A two-way ANOVA with repeated measures showed a significant effect of time (*F*_(3,108)_ = 136.95, *P* < 0.01), treatment (*F*_(3,36)_ = 1.615, *P* = 0.203) and time × treatment interaction (*F*_(9,108)_ = 22.927, *P* < 0.01) among these groups. Furthermore, Newman-Keuls *post hoc* test revealed that the endurance time of the histamine group (*n* = 10) on the rota-rod at 0 h after microinjection significantly increased compared with that of the saline group (*n* = 10; *P* < 0.01, Figure 2A), and such effect recovered hours later (Figure 2A). Activation of histamine H2 receptors in LVN by microinjection of dimaprit (*n* = 10) mimicked the histamine-induced improvement in motor performances at 0 h after injection (*P* < 0.05, Figure 2A). And blockage of histamine H2 receptors in the LVNs by ranitidine (*n* = 10) remarkably shortened the endurance time of rats on the rotating rod at 4 h (*P* < 0.05, Figure 2A) and 24 h (*P* < 0.05, Figure 2A) after injection.

In the balance beam test, the mean score of 40 rats of all groups on the beam was 4.46 ± 0.06 s, and there was no significant difference among the groups before injections (*F*_(3,36)_ = 0.142, *P* = 0.934; Figure 2B). A two-way ANOVA with repeated measures revealed a significant effect of time (*F*_(3,108)_ = 151.091, *P* < 0.01), treatment (*F*_(3,36)_ = 7.626, *P* < 0.01) and time × treatment interaction (*F*_(9,108)_ = 36.534, *P* < 0.01) among these groups. Furthermore, Newman-Keuls *post hoc* test indicated that microinjection of histamine (*n* = 10) into the LVNs significantly shortened the time that traversing the balance beam at 0 h after injection compared with the saline group (*n* = 10; *P* < 0.01, Figure 2B), and such effect recovered hours later (Figure 2B). Notably, at 0 h after injection, the spending time of the dimaprit group (*n* = 10) was significantly shorter than that of the saline group (*P* < 0.01, Figure 2B), whereas the time traversing the beam of the ranitidine group (*n* = 10) markedly prolonged at 4 h, even 24 h after injection compared with that of the saline group (*P* < 0.01, Figure 2B). These results indicate that activation of histamine H2 receptors in the LVNs promotes animal’s motor balance and coordination.

### HCN Channels and K^+^ Channels Are Involved in the Histamine-Induced Excitation on LVN Neurons

**Figure 3:**
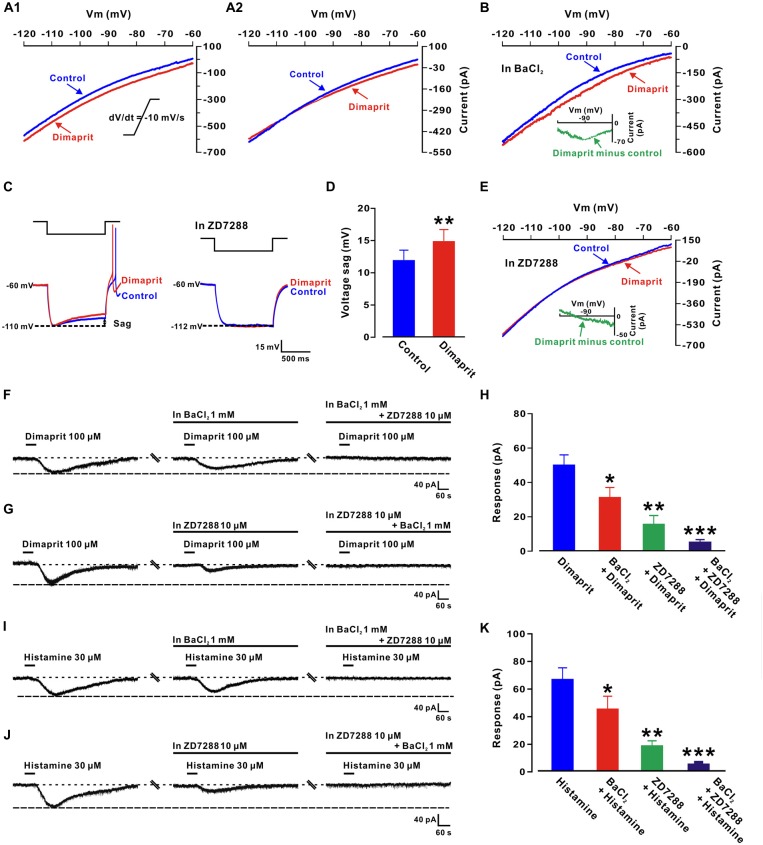
**Hyperpolarization-activated cyclic nucleotide-gated (HCN) channels and K^+^ channels co-mediate the excitation induced by activation of H2 receptors on LVN neurons. (A1,A2)** Two types of dimaprit-induced changes of I-V curves on LVN neurons (*n* = 9 and 2, respectively) responding to a slow-ramp command (dV/dt = −10 mV/s). The diversity of the dimaprit-induced changes in I-V relationships suggests that more than one ionic basis is involved in histamine H2 receptor-mediated inward current on LVN neurons. The intersection of I-V curves at calculated *E*_k_ of −108 mV **(A2)** on 18.2% (2/11) of neurons indicates an involvement of K^+^ channels in the H2 receptor-mediated LVN neuronal excitation. **(B)** In the ACSF containing Ba^2+^, a blocker of K^+^ channels, the dimaprit-induced changes of I-V curves and the current excluding K^+^ component in slow-ramp command tests. Note that in the presence of Ba^2+^, the dimaprit-induced current (the inset) showed a significant feature of hyperpolarization activation, which is consistent with the characteristics of current of HCN channels. **(C)** Inward rectification (sag) triggered by hyperpolarizing current steps on an LVN neuron was increased by dimaprit (the left panel). ZD7288, a highly selective HCN channel antagonist, totally blocked the increase in the sag induced by dimaprit (the right panel).** (D)** Group data of the tested LVN neurons. **(E)** In the slow-ramp command test, in the presence of ZD7288 to exclude the component of HCN channel current, the dimaprit-induced residual current (the inset) was very small and reverted near the calculated *E*_k_. **(F)** BaCl_2_ partly blocked the dimaprit-elicited inward current, and combined application of BaCl_2_ and ZD7288 totally blocked the current. **(G)** The dimaprit-induced inward current was also partly blocked by ZD7288, and totally blocked by combined application of ZD7288 and BaCl_2_. **(H)** Group data of the tested LVN neurons. **(I,J)** Histamine-induced inward current was partly/totally blocked by separate/combined application of ZD7288 and BaCl_2._ **(K)** Group data of the tested LVN neurons. Data shown are means ± SEM; *P < 0.05, **P < 0.01, ***P < 0.001.

To clarify the ionic mechanisms underlying the excitation of LVN neurons elicited by H2 receptor activation, a slow-ramp command test was employed to assess the dynamic features of dimaprit-induced current. As shown in Figures 3A1,A2, two types of the I-V curves induced by dimaprit were observed, indicating that more than one ionic mechanism may be underlying the depolarization induced by the activation of H2 receptors on LVN neurons. Notably, the I-V curves of 18.2% (2/11) recorded LVN neurons intersected at −110 mV (Figure 3A2), which means the dimaprit-elicited inward current reverses near the calculated *E*_k_ of −108 mV, suggesting an involvement of K^+^ channels. Moreover, Ba^2+^, a blocker of K^+^ channels (McCormick and Williamson, 1991), was applied to examine the dynamic properties of the dimaprit-induced current excluding the component of potassium. As shown in Figure 3B, only one change in the I-V curves was observed after blocking K^+^ current. Subtracting the control from the current recorded during dimaprit application yielded a difference current representing the dimaprit-induced current excluding the K^+^ component (the insert panel in Figure 3B). The difference current showed a significant feature of hyperpolarization activation, which is in line with the characteristics of the current of HCN channels. Since depolarizing voltage sag induced by hyperpolarizing current steps was one of the hallmarks of HCN channel activation (Pape, 1996), we further observed the effect of dimaprit on voltage sag on LVN neurons and found that the sag was remarkably increased by dimaprit (from 11.8 ± 1.6 mV to 14.6 ± 1.9 mV, *n* = 6, *P* < 0.01; Figures 3C,D). Furthermore, after blockage of HCN channels with ZD7288 (10 μM), a selective blocker for HCN channels, the HCN channel activation-induced sag vanished and the increase of voltage sag elicited by dimaprit was totally blocked (Figures 3C,D). The results, together with the dynamic properties of hyperpolarization activation observed in slow-ramp command test after excluding the component of K^+^, strongly suggests that HCN channels participate in the mediation of excitation of LVN neurons induced by the activation of H2 receptors. On the other hand, the dimaprit induced I-V curve changes were also detected in the presence of ZD7288 in the slow-ramp command test (Figure 3E). As shown in the insert panel of Figure 3E, the dimaprit-induced current excluding the component of HCN channel current reverted near the calculated *E*_k_. Although the residual K^+^ current was quite small, the data indicate a co-mediation of K^+^ and HCN channels in the dimaprit-induced excitation on LVN neurons. In addition, we found that separate application of BaCl_2_ or ZD7288 partially inhibited the dimaprit-elicited inward current (50.2 ± 5.8 pA, *n* = 11) to 31.3 ± 5.7 pA (*n* = 6, *P* < 0.05, Figures 3F,H) and 16.1 ± 4.7 pA (*n* = 5, *P* < 0.01, Figures 3G,H), respectively, whereas combined application of BaCl_2_ and ZD7288 totally blocked the current induced by dimaprit (*n* = 10, *P* < 0.001, Figures 3F–H). Similarly, as shown in Figures 3I–K, the histamine-induced inward current (67.4 ± 8.2 pA, *n* = 11) on LVN neurons was also partly blocked by separate application of BaCl_2_ or ZD7288 to 46.0 ± 9.3 pA (*n* = 6, *P* < 0.05) or 18.8 ± 3.6 pA (*n* = 5, *P* < 0.001), respectively, and totally blocked by combined application of BaCl_2_ and ZD7288 (*n* = 9, *P* < 0.001). All these results strongly suggest that a dual ionic mechanism, involving both the activation of HCN channels and the closure of K^+^ channels, may mediate the excitatory effect of activation of H2 receptors on LVN neurons.

### Activation of HCN Channels Coupled to H2 Receptors Increases Sensitivities of LVN Neurons and Changes their Dynamic Properties

**Figure 4:**
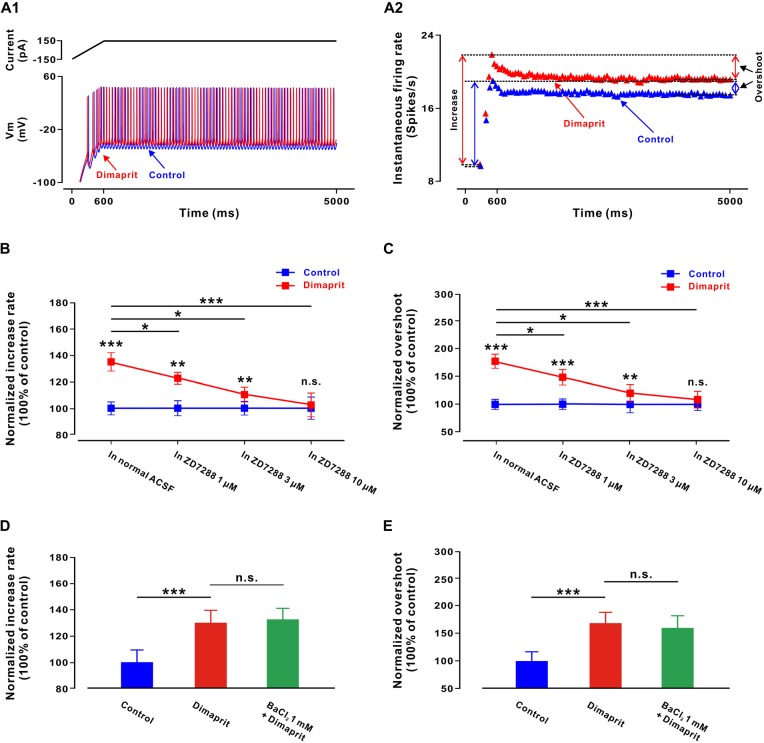
**Activation of HCN channels coupled to H2 receptors increases sensitivity of LVN neurons and change their dynamic properties. (A1)** A ramp-like current was given from a hyperpolarized level of −150 pA for 600 ms to reach +150 pA, with the final steady-state value of +150 pA lasting 4400 ms. Firing response of a LVN neuron to the ramp-like current stimulation in the absence and presence of dimaprit. **(A2)** Instantaneous firing rates of the same LVN neuron to the ramp-like current in the absence and presence of dimaprit showed that H2 receptor activation remarkably raised both the rate of increase of the instantaneous firing rate (spikes/s/nA) and the overshooting response. **(B)** The normalized enhancement in the rate of increase in the instantaneous firing rate was concentration-dependently blocked by ZD7288 (1, 3 and 10 μM), indicating that the dimaprit-induced increment in sensitivity of LVN neurons was mediated by the activation of HCN channels coupled to H2 receptors. **(C)** ZD7288 (1, 3 and 10 μM) concentration-dependently blocked the dimaprit-induced overshoot increment, suggesting that the activation of HCN channels coupled to H2 receptors also contributed to the changes of dynamic properties of LVN neurons. **(D,E)** BaCl_2_ (1 mM) did not block the dimaprit-induced enhancement in the rate of increase in the instantaneous firing rate and increment in overshoot on five LVN neurons. Data shown are means ± SEM; *P < 0.05, **P < 0.01 and ***P < 0.001 and n.s. indicates non-significant.

Intriguingly, besides increasing the excitability, the activation of H2 receptors also enhanced the sensitivity of the LVN neurons to the stimulation of a depolarizing ramp-like current (Figures 4A1,A2), which consists of a 600 ms ramp (from −150 pA to 150 pA, slope of 0.5 nA/s) followed by a long plateau of current (150 pA, 4400 ms; Ris et al., 2001; Uno et al., 2003; Zhang et al., 2011). On five LVN neurons, dimaprit (100 μM) significantly increased the rate of increase in the instantaneous neuronal firing rate (from 100.0 ± 5.1% to 135.2 ± 6.9%, *n* = 5, *P* < 0.001; Figure 4B). However, in the presence of ZD7288 (1, 3 and 10 μM), as illustrated in Figure 4B, the enhancement in the rate of increase in the instantaneous firing rate induced by dimaprit (100 μM) was blocked in a concentration dependent manner. The normalized enhancement in the rate of increase in the instantaneous firing rate was decreased significantly from 35.2 ± 6.9% to 21.4 ± 4.2% (*n* = 5, *P* < 0.05), 10.0 ± 5.4% (*n* = 5, *P* < 0.05) and 2.6 ± 8.9% (*n* = 5, *P* < 0.001), respectively (Figure 4B). The results indicate that the increment in sensitivity of LVN neurons induced by the activation of histamine H2 receptors is mediated by HCN channels.

We also measured the difference between the instantaneous firing rate reached at the end of the ramp and the stable discharge rate at the end of the plateau, i.e., the overshoot (Figure 4A2). This parameter reflects the nonlinear, dynamic properties of neurons. We found that dimaprit (100 μM) effectively increased the overshoot of the LVN neurons from 100.0 ± 8.1% to 179.6 ± 12.5% (*n* = 5, *P* < 0.001; Figure 4C), and ZD7288 (1, 3 and 10 μM) concentration-dependently blocked the dimaprit-induced increment in overshoot from 79.6 ± 12.5% to 48.6 ± 13.2% (*n* = 5, *P* < 0.05), 20.5 ± 16.1% (*n* = 5, *P* < 0.05) and 8.9 ± 14.8% (*n* = 5, *P* < 0.001), respectively (Figure 4C). Therefore, the activation of HCN channels coupled to H2 receptors not only increases sensitivity of LVN neurons, but also changes their dynamic properties.

Besides activation of HCN channels, closure of K^+^ channels is the other ionic mechanism underlying the activation of H2 receptors on LVN neurons. Thus, the dimaprit-induced changes in sensitivities of LVN neurons and their dynamic properties were also measured in the presence of BaCl_2_. However, we found that BaCl_2_ (1 mM) blocked neither the dimaprit-induced enhancement in the rate of increase in the instantaneous firing rate (from 30.4 ± 6.3% to 32.6 ± 5.5%, *n* = 5, *P* = 0.64, Figure 4D), nor the increment in overshoot (from 66.9 ± 20.2% to 60.1 ± 23.1%, *n* = 5, *P* = 0.48, Figure 4E). Therefore, it is HCN channels, but not K^+^ channels coupled to H2 receptors, that contribute to the modulation on sensitivity and dynamic properties of LVN neurons by the activation of H2 receptors. We speculate that HCN channels may actively participate in the LVN-mediated motor behaviors.

### Blockage of HCN Channels Coupled to H2 Receptors in the LVN Attenuates Motor Balance and Coordination

**Figure 5:**
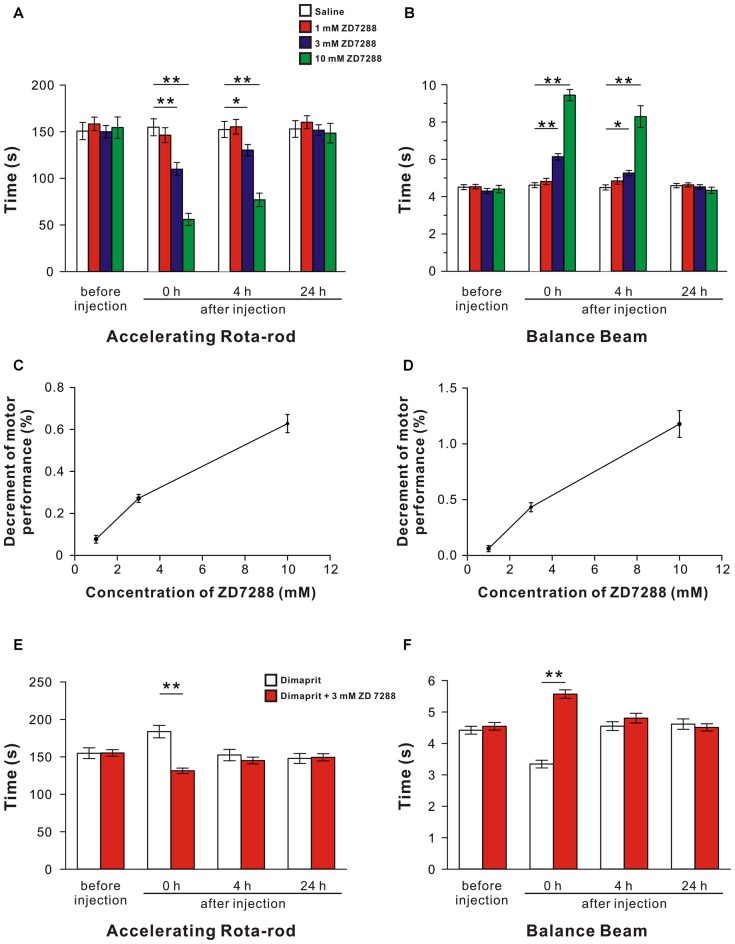
**Blockage of HCN channels coupled to H2 receptors in the LVN attenuates spontaneous and the dimaprit-induced promotion on motor balance and coordination. (A,B)** The endurance time on an accelerating rota-rod and the duration of passage through the balance beam of rats treated by bilateral microinjection of saline, 1 mM ZD7288, 3 mM ZD7288 or 10 mM ZD7288. **(C,D)** The decrement of motor performance in accelerating rota-rod and balance beam tests increased with the increase of the concentration of ZD7288. **(E,F)** The endurance time on an accelerating rota-rod and the duration of passage through the balance beam of rats treated by bilateral microinjection of dimaprit or dimaprit together with 3 mM ZD7288. Data shown are means ± SEM; *P < 0.05, **P < 0.01.

Given that the effects of HCN channels’ activation on both sensitivity and dynamic properties of LVN neurons *in vitro*, ZD7288 (1 mM, 3 mM and 10 mM), or dimaprit together with ZD7288, was microinjected into bilateral LVNs to examine the role of HCN channels in the histamine H2 receptor-mediated promotion in motor behaviors on accelerating rota-rod and balance beam. For the accelerating rota-rod test, no significant difference among the groups before injections was observed (*F*_(3,36)_ = 0.19, *P* = 0.904; Figure 5A). A significant effect of time (*F*_(3,108)_ = 90.146, *P* < 0.01), treatment (*F*_(3,36)_ = 8.291, *P* < 0.01) and time × treatment interaction (*F*_(9,108)_ = 34.988, *P* < 0.01) among these groups was revealed by two-way ANOVA with repeated measures. Furthermore, *post hoc* test showed that microinjection of 3 or 10 mM ZD7288 (*n* = 10) into the LVNs remarkably decreased the endurance time of rats on the rotating rod at 0 h and 4 h after injection compared with the rats microinjected with saline (*n* = 10; *P* < 0.01, *P* < 0.05, Figure 5A), and such effect recovered hours later (Figure 5A). For the balance beam test, no significant difference was found among the groups before injections (*F*_(3,36)_ = 0.497, *P* = 0.687; Figure 5B). And a significant effect of time (*F*_(3,108)_ = 92.131, *P* < 0.01), treatment (*F*_(3,36)_ = 45.458, *P* < 0.01) and time × treatment interaction (*F*_(9,108)_ = 42.286, *P* < 0.01) among these groups was determined. Furthermore, *post hoc* test revealed that microinjection of 3 or 10 mM ZD7288 (*n* = 10) into the LVNs concentration-dependently lengthened the spent traversing time of rats on the beam at 0 h and 4 h after injection compared with the saline group (*n* = 10; Figure 5B), and such effect recovered hours later (Figure 5B). These results indicate that blockage of HCN channels coupled to histamine H2 receptors attenuates motor balance and coordination on the rota-rod and balance beam.

Moreover, the decrement of motor performance in both accelerating rota-rod (Figure 5C) and balance beam (Figure 5D) tests increased with the increase of the concentration of ZD7288. Also, it is noteworthy that the improvement of motor performances on rota-rod and balance beam induced by activation of H2 receptors by dimaprit was remarkably blocked by ZD7288 (*P* < 0.01, respectively; Figures 5E,F). All these results strongly suggest that HCN channels coupled to H2 receptors mediate improvement in motor balance and coordination of histaminergic inputs.

## Discussion

Although the central histaminergic system solely originates from the tuberomammillary nucleus of the hypothalamus, it participates in the regulation of various basic physiological functions (Haas et al., 2008). Brain histamine depletion or knockout of histamine receptor reduced animals’ locomotor activity and exploratory behavior (Onodera et al., 1994; Inoue et al., 1996; Toyota et al., 2002), indicating the central histaminergic system may also hold a critical position in somatic motor functions. Yet, the functional role of histaminergic system in various motor structures and the underlying mechanism is still little known. Here, in the present study, we report that histamine H2 receptor and its coupled HCN channel mediate the histamine-induced enhancement of LVN neuronal excitability and sensitivity. Via activation of histamine H2 receptors and the downstream HCN channels, histaminergic inputs promote the LVN-mediated motor behaviors.

### Electrophysiological and Behavioral Effects of Histamine in the LVN

Immunohistochemical studies have demonstrated that the histaminergic neurons in the tuberomammillary nucleus of the hypothalamus project directly to the vestibular nuclei in brainstem (Schwartz et al., 1991; Steinbusch, 1991; Tighilet and Lacour, 1996). As an essential part of the central histaminergic system, these histaminergic innervations on the vestibular nuclear complex, especially their physiological functions, have received increasing attention. Intriguingly, histamine exerts a uniformly excitatory effect on all four vestibular sub-nuclei (Wang and Dutia, 1995; Zhang et al., 2008, 2013; Peng et al., 2013; Zhuang et al., 2013; Yu et al., 2016). However, the receptor mechanisms underlying the histamine-induced excitation on these sub-nuclei are various (Wang and Dutia, 1995; Zhang et al., 2008, 2013; Peng et al., 2013; Zhuang et al., 2013; Yu et al., 2016). Histamine H1 and H2 receptors co-mediate the excitatory effect of histamine on neurons in the superior and inferior vestibular nuclei (Peng et al., 2013; Zhuang et al., 2013; Yu et al., 2016), whereas histamine H1, H2 and H3 receptors are all involved in the complex modulation of histamine on medial vestibular nucleus (MVN) neuron (Wang and Dutia, 1995; Bergquist and Dutia, 2006; Zhang et al., 2013). In this study, we found that histamine elicited a significant excitatory response on LVN neurons both having spontaneous firing and being silent. These histamine-elicited excitations were totally blocked by ranitidine (selective histamine H2 receptor antagonist) and mimicked by dimaprit (highly selective agonist for histamine H2 receptors), suggesting that only histamine H2 receptors mediate the excitatory effect of histamine on LVN neurons.

Among the four vestibular sub-nuclei, the LVN receives inputs from the semicircular canals and the utricle, and projects into the lateral vestibulo-spinal tract to ipsilaterally innervate the ventral horn of the spinal cord (Carleton and Carpenter, 1983; Sarkisian, 2000). Through facilitating the activity of spinal motoneurons innervating gravity-opposing muscles of the limb, the LVN is actively involved in the vestibule-spinal reflex and postural control. Thus, it is naturally speculated that the excitatory modulation of histamine on LVN neurons via H2 receptors may enhance the output of LVN, excite the neurons of their targets, and influence motor behaviors. In the present study, our results demonstrate that microinjection of histamine into the LVN significantly promotes motor performances on accelerating rota-rod and balance beam. Activation of histamine H2 receptors mimics the histamine-induced promotion in motor performances, whereas blockage of H2 receptors to block endogenous histaminergic inputs into the LVN attenuates motor behaviors. These results suggest that histamine H2 receptors contribute to the improvement of histaminergic inputs in the LVN-mediated motor behaviors. Interestingly, our previous studies found that histamine promoted motor balance and coordination via the activation of H2 receptors in the fastigial nucleus (He et al., 2012) and interpositus nucleus (Song et al., 2006) of the cerebellum. Therefore, histamine H2 receptors may hold a critical position in central histaminergic modulation on motor control.

### Ionic Mechanisms Coupled to Histamine H2 Receptor in the LVN

Several types of ionic channels/exchangers have been reported to be linked to histamine receptors and modulate the excitability of central vestibular nuclear neurons (Ris et al., 2001; Zhang et al., 2013, 2016; Yu et al., 2016). Here, we find that both the HCN channels and K^+^ channels are involved in the excitation of LVN neurons induced by the activation of histamine H2 receptors. In these dual ionic mechanisms, HCN channels seem to play a major contribution, whereas K^+^ component may account for only a small one. Since HCN channels are critical “pacemaker channels” of neurons (Pape, 1996) and K^+^ channels are responsible for setting membrane potential, histamine released from the hypothalamus will help to accelerate membrane depolarization and the generation of LVN neuronal activity via opening of HCN channels and closure of K^+^ channels. Notably, in this study, we find that HCN channels, but not K^+^ channels coupled to H2 receptors, are responsible for the histamine-induced enhancement of sensitivities of LVN neurons and modulation of their dynamic properties. This may be owing to the fact that HCN channels play an essential role in not only governing neuronal excitability but also controlling the way that neurons respond to input. Thus, by activation of HCN channels coupled to H2 receptors, histaminergic inputs may enhance the sensitivity and responsiveness of LVN neuronal circuitry to periphery vestibular inputs and consequently modulate LVN-mediated motor behaviors.

Dysfunction of HCN channels is usually associated with pathological conditions, such as epilepsy, age-related working memory decline and neuropathic pain (Lewis and Chetkovich, 2011; He et al., 2014). In the present study, by means of pharmacological manipulation to block HCN channels in the LVN, we find that rat motor performances on the rota-rod and balance beam remarkably decline (Figures 5A–D) and the improvement of motor performances induced by activation of histamine H2 receptors is significantly abolished (Figures 5E,F). These results, together with our previous findings that blockage of HCN channels in the cerebellar nuclei attenuates motor performances (Zhang et al., 2016), suggest that HCN channels coupled to histamine H2 receptors may mediate the modulation of the central histaminergic system on motor behaviors and play an important role in somatic motor control.

### Functional Significance of Histaminergic Innervation on the LVN

Besides histaminergic innervation, the LVN also receives excitatory orexinergic inputs from the hypothalamus (Zhang et al., 2011). Unlike the critical role of orexinergic modulation in motor challenge (Zhang et al., 2011), excitatory histaminergic inputs on the LVN may be responsible for routine execution of normal function of the central vestibular nuclear circuitry. The functional difference in aspect of LVN’s motor control between the histaminergic and orexinergic systems may depend on different functional roles of the origins of these two systems. In conclusion, the present study shows that the histamine-elicited excitation on the LVN neurons is mediated by postsynaptic histamine H2 receptor and its downstream HCN channels and K^+^ channels. The activation of HCN channels coupled to H2 receptors increases sensitivity of LVN neurons, and improves the LVN-mediated motor behaviors. Based on these results, we suggest that via histamine H2 receptors and the downstream HCN channels, the excitatory histaminergic inputs may modulate both excitability and sensitivity of the LVN neurons and are actively involved in the central vestibular postural and motor control.

## Author Contributions

BL and X-YZ performed experiments, analyzed data, and prepared figures and drafts. A-HY, X-CP, Z-PC and J-YZ performed some experiments. Y-SC discussed the research. J-NZ and J-JW designed research and wrote the article.

## Funding

This work was supported by the National Natural Science Foundation of China (grants 31330033, 91332124, 31471112, 31500848, 81671107, 31600834, J1210026 and NSFC/RGC Joint Research Scheme 31461163001); the Ministry of Education, China (SRFDP/RGC ERG grant 20130091140003, and Fundamental Research Fund for the Central Universities 20620140542 and 020814380048); the Natural Science Foundation of Jiangsu Province, China (grant BK20140599); and the China Postdoctoral Sciences Foundation (grant 2013T60520).

## Conflict of Interest Statement

The authors declare that the research was conducted in the absence of any commercial or financial relationships that could be construed as a potential conflict of interest.
